# Supplementary material for: STEFTR: A Hybrid Versatile Method for State Estimation and Feature Extraction From the Trajectory of Animal Behavior
Source: Front Neurosci. 2019 Jun 28;13:626. doi: 10.3389/fnins.2019.00626 (PMC6611002; doi:10.3389/fnins.2019.00626)
Supplement: Supplementary file 2 [file Data_Sheet_1.PDF]

Suppl. Figure 1

A

Predicted	Actual	
cluster	run	pir.
**0**	**41,137**	1,950
**1**	3,291	**18,738**
**2**	339	**13,236**
**3**	62	**6,956**
**4**	1	**2,091**

B

Predicted	Actual	
cluster	run	pir.
**0**	**40,099**	11,298
**1**	3,150	**14,727**
**2**	1,409	**15,092**

C

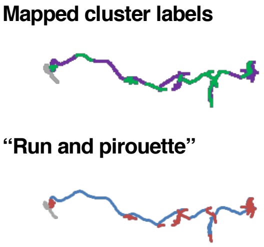

D

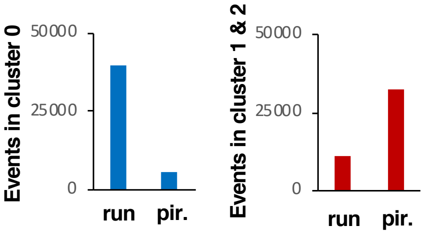

E

Predicted	Actual	
cluster	run	pir.
**0**	2,778	2,625
**1**	10,826	6,917
**2**	21,607	21,182
**3**	7,342	10,143
**4**	2,858	2,811

Supplementary Figure 1. State estimation of worms (Related to Figure 2). (A) Confusion matrix for the state estimation with *dB_Var*. (B-D) The results of state estimation with *dV_Var* in confusion matrix, where the histogram was separated to 3 clusters. (B) Confusion matrix. (C) Mapping on a trajectory. (D) Event numbers of run and pirouette in cluster 0 (left) and 1 & 2 (right). (E) Confusion matrix for the state estimation by *dB_Var* in finer (0.15%) window.

Suppl. Figure 2

A

Predicted	Actual			
**cluster**	**rest**	**walk**	**swim**	**dive**
**0**	**12,177**	212	-	-
**1**	152	**1,763**	-	-
**2**	125	**2,743**	561	963
**3**	1	166	**1,304**	**1,210**
**4**	-	2	**2,691**	190

B

Predicted	Actual			
**cluster**	**rest**	**walk**	**swim**	**dive**
**0**	8,861	1,225	189	-
**1**	3,510	1,278	71	-
**2**	321	2,544	3,766	2,205
**3**	-	46	441	157
**4**	-	4	203	13

C

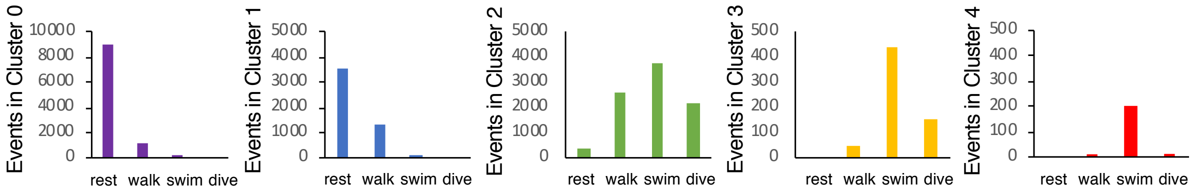

D

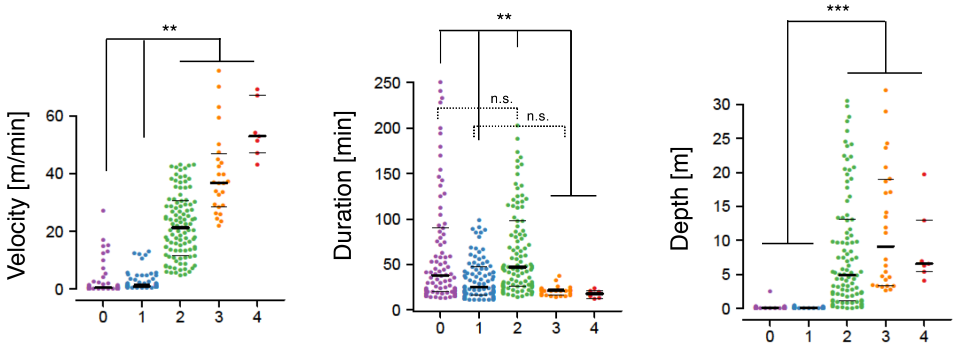

Supplementary Figure 2. State estimation of penguins (Related to Figure 3). (A) Confusion matrix for the state estimation with *dV_Ave*. (B-D) The results of state estimation with *dV_Ave* in confusion matrix (B) and event numbers of each cluster (C). (D) Differences in velocity, duration, and depth among the clusters with *dV_Ave*. Each dot represents a cluster bout, and the bars represent the median and the first and third quartiles. Statistical values were calculated using Kruskal-Wallis test with *post hoc* Dunn's test. $^{**}p < 0.01$, $^{***}p < 0.001$.
